# 1.5 GPa Grade High-Strength Steel Sheet Flattening by Roll Gap Adjustment Considering Pattern Roll Effects

**DOI:** 10.3390/ma18081702

**Published:** 2025-04-09

**Authors:** Youngjin Jeon, Kyucheol Jeong, Geun-ho Kim, Jonghun Yoon

**Affiliations:** 1 Department of Mechanical Design Engineering, Hanyang University, 55, Hanyangdaehak-ro, Sangrok-gu, Ansan 15588, Gyeonggi, Republic of Korea; jj981226@hanyang.ac.kr (Y.J.); kcjeong0@gmail.com (K.J.); kdh7355@secoasan.com (G.-h.K.); 2 2BK21 FOUR ERICA-ACE Center, Hanyang University, Ansan 15588, Gyeonggi, Republic of Korea; 3 ASAN Co., Ltd., Pureundeulpan-ro 826-4, Paltan-Myun, Hwasung 18525, Gyeonggi, Republic of Korea

**Keywords:** advanced high-strength steel, flattening, finite element analysis, roll gap optimization

## Abstract

This study analyzes a three-stage roll flattening process to improve the flatness of 1.5 GPa grade AHSS sheets. Unlike conventional leveler rolls, which mainly relieve residual stress through longitudinal tension-compression, the second roll has a sloped pattern to induce transverse deformation and redistribute local residual stresses. A twisted sheet was processed under different roll gap settings (1.3 mm, 1.1 mm, 0.9 mm, and 0.7 mm), and experimental measurements were compared with Abaqus Explicit simulations. At a 1.1 mm gap, the RMSE between experiment and simulation is 0.22 mm, showing the highest agreement. Both twist and crossbow defects are reduced by over 80%, achieving optimal flattening. At 1.3 mm, the simulation overestimates the second roll’s effect, causing excessive localized deformation. Reducing the gap to 0.9 mm or 0.7 mm increases discrepancies due to roll fixation differences. Experiments allow more central bending, amplifying crossbow, while simulations assume rigid rolls, underestimating curvature. Adjusting the second roll’s geometry to enhance transverse tension-compression and setting the gap to 1.1 mm effectively reduces defects. This method improves flatness while minimizing the number of rolls needed in high-strength steel sheet production.

## 1. Introduction

In modern industries, metal sheets are processed through various methods, including roll forming, to create essential components for automotive, aerospace, and electronic applications. In particular, the automotive and aerospace sectors have increasingly adopted Advanced High Strength Steel (AHSS) due to its high strength and lightweight properties, which contribute to improved fuel efficiency and enhanced safety during collisions [1]. To clarify the application of AHSS in the automotive industry, we have explicitly described its use in structural components such as B-pillars, door rings, front bumper beams, subframes, and suspension arms. Furthermore, in electric vehicle (EV) manufacturing, AHSS plays a crucial role in battery protection structures and crash-resistant designs, ensuring both structural integrity and weight reduction. However, during the coiling and uncoiling processes of AHSS, residual stress is generated, leading to potential defects that may negatively affect the quality of the final products [2,3]. 

**Figure 1:**
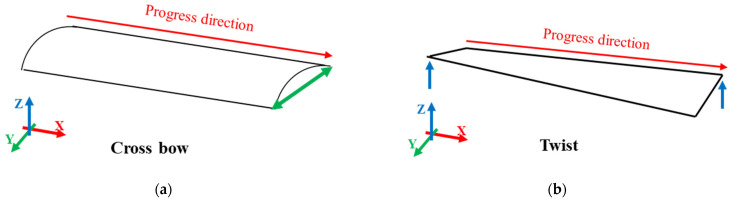
Sheet metal defects: (**a**) crossbow defect causing curvature in the width direction; (**b**) twist defect resulting in combined longitudinal and transverse distortions.

These defects typically appear in various forms depending on the distribution of stress within the sheet. Figure 1 illustrates two common types of distortions observed in sheet metal. Figure 1a shows a crossbow defect, where the edges of the sheet bow upward or downward, creating an arched surface across its width. Figure 1b shows a twist defect, which combines both longitudinal and transverse distortions, resulting in a spiraling deformation that makes the sheet challenging to process further. To minimize such defects, flattening processes are required to improve the flatness and quality of the sheets.

The leveler roll is a widely used and essential technology for improving the flatness of metal sheets by reducing residual stress through repetitive bending and relaxation processes. This process allows for the uniform distribution of residual stress within the material, enhancing its flatness and improving the overall surface quality [4,5]. During flattening, the metal sheet undergoes both plastic strain and elastic recovery, which optimizes its physical properties and contributes to producing high-quality final products [6].

However, the leveler roll typically requires more than 10 rolls for flattening processes [4,5,6,7]. This is a necessary design element to improve the quality of flattening, but it also leads to an increase in energy consumption. As the number of rolls increases, additional energy is consumed due to the rotation of each roll and its interaction with the material, which subsequently results in higher operating costs in industrial processes [6,8]. Furthermore, as the number of rolls increases, the structural complexity of the equipment grows, making maintenance and roll replacement more challenging. During roll replacement, adjustments to the arrangement and spacing of the rolls are required, which can prolong the process and negatively impact production efficiency [9]. In conclusion, while the leveler roll is an essential technology for flattening metal sheets, the increase in the number of rolls leads to a more complex process and extended processing time, which are significant drawbacks [9,10].

This study proposes a novel roll flattening method that enhances the flatness of metal sheets while reducing the number of leveling rolls. Unlike conventional levelers that rely on multiple rolls to relieve residual stress through repeated longitudinal deformation, the proposed three-stage system optimizes roll gap and geometry to achieve comparable or superior flattening performance with only three rolls. A key innovation is the introduction of a slope-patterned second roll, which induces transverse deformation and enables local redistribution of residual stress across the sheet width—an effect that conventional methods cannot achieve. 

This reduction in roll count brings significant industrial benefits, including simplified equipment design, lower manufacturing costs, and reduced energy consumption due to decreased rotational resistance. 

To validate the effectiveness of the method, both experimental tests and finite element analysis (FEA) were conducted under roll gap settings of 1.3 mm, 1.1 mm, 0.9 mm, and 0.7 mm. Flattening performance was evaluated by calculating the root mean square error (RMSE) between experimental and simulation results, considering shape defects such as crossbow and twist. Factors like contact pressure distribution, friction, and dynamic effects were also analyzed to identify discrepancies. Based on these results, an optimal roll gap was determined that minimizes defects and shows strong agreement between experiments and simulations.

## 2. Experimental Procedure

### 2.1. Materials

The material used in this study is martensitic steel for cold forming with an ultimate tensile strength of 1.5 GPa. It undergoes quenching and tempering during manufacturing, which ensures high strength and adequate ductility. Therefore, no additional annealing heat treatment was performed before cold working in this study.

**Figure 2:**
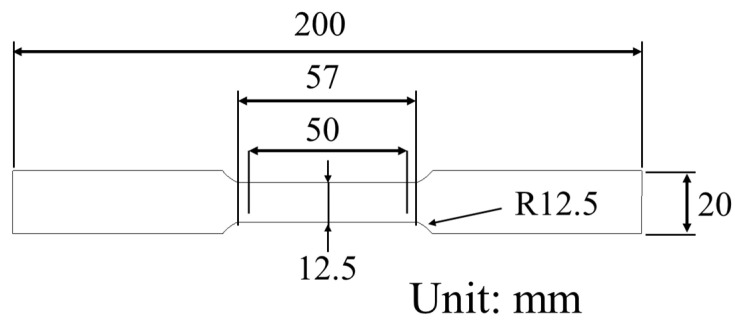
Dimension of test specimen.

**Figure 3:**
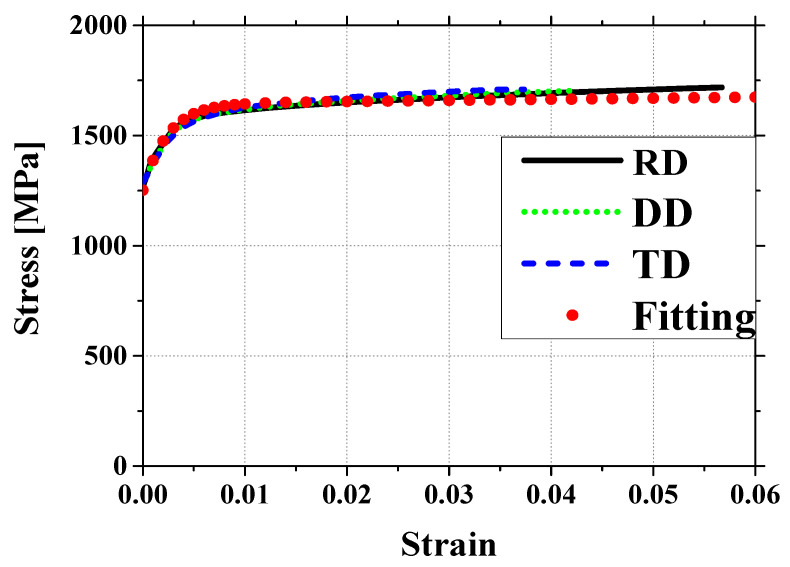
Stress–strain curves for martensitic steel in RD, DD, and TD directions with fitted results.

To evaluate its mechanical properties and anisotropy, tensile tests were conducted on specimens cut in three different directions: rolling direction (RD), diagonal direction (DD), and transverse direction (TD). The dimensions of the test specimens are presented in Figure 2, while the stress–strain curves obtained from these experiments are shown in Figure 3. The results reveal minimal differences in mechanical behavior between RD, DD, and TD, indicating that the material exhibits nearly isotropic behavior [11,12,13]. The sample preparation and testing followed the ASTM E8 tensile test standard, which specifies guidelines for specimen geometry, dimensions, and testing procedures to ensure accurate evaluation of mechanical properties.

Several studies have demonstrated that although some initial anisotropy may exist in martensitic steels, the hot-forming and quenching process significantly reduces directional differences in mechanical properties. As the microstructure transforms into fine-grained martensite, anisotropic effects diminish, making an isotropic material model a reasonable choice for simulations without significant loss of accuracy [11,12,13,14]. Additionally, employing an anisotropic model significantly increases computational time, and its practical effectiveness must be carefully considered. Based on the tensile test results in this study, the mechanical properties of martensitic steel after press hardening exhibit minimal directional differences, confirming that assuming isotropic behavior in simulations does not significantly affect reliability. Therefore, an isotropic material model was applied in this study to enhance computational efficiency while maintaining accuracy within acceptable limits.

The stress–strain curves were fitted using the Voce hardening model, which is widely used to describe the plastic behavior of metals and accounts for both initial yield stress and subsequent strain hardening [15]. The Voce model is expressed as

(1) $$\sigma \left(\varepsilon_{p}\right)=\sigma_{y}+n_{1}\cdot p\left[1-exp\left(-n_{2}\varepsilon_{p}\right)\right]+n_{3}\varepsilon_{p},$$

where $\sigma \left(\varepsilon_{p}\right)$ is the reference flow stress, and $\sigma_{y},\;n_{1},\;n_{2},\;n_{3}$ are material parameters calibrated from the tensile test data. This model is typically applied to true stress-true strain data, as it effectively captures strain hardening beyond yielding, making it more suitable for describing plastic deformation compared to engineering stress–strain data. Additionally, the Young’s modulus (E) and Poisson’s ratio (ν) were also obtained and are presented in Table 1.

### 2.2. Experimental Setup

**Figure 4:**
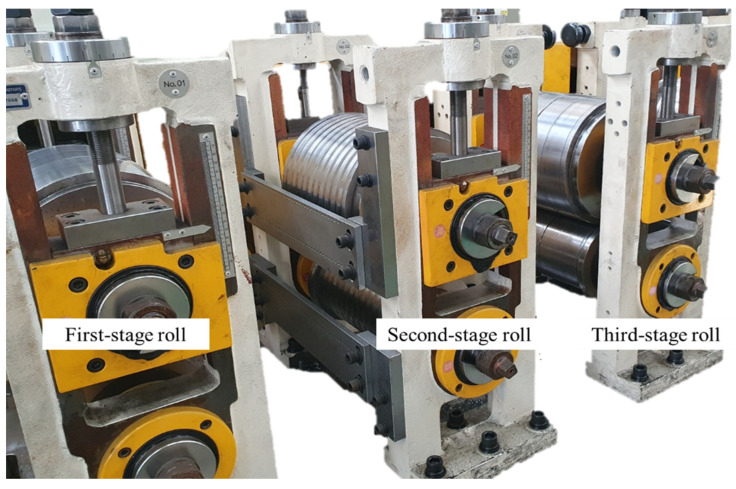
Experimental setup.

The experiment was conducted using a roll flattening machine equipped with a three-stage roll configuration. Figure 4 illustrates the overall setup of the experimental equipment. The machine consists of a first-stage roll, a second-stage roll, and a third-stage roll, each specifically designed for its unique purpose.

**Figure 5:**
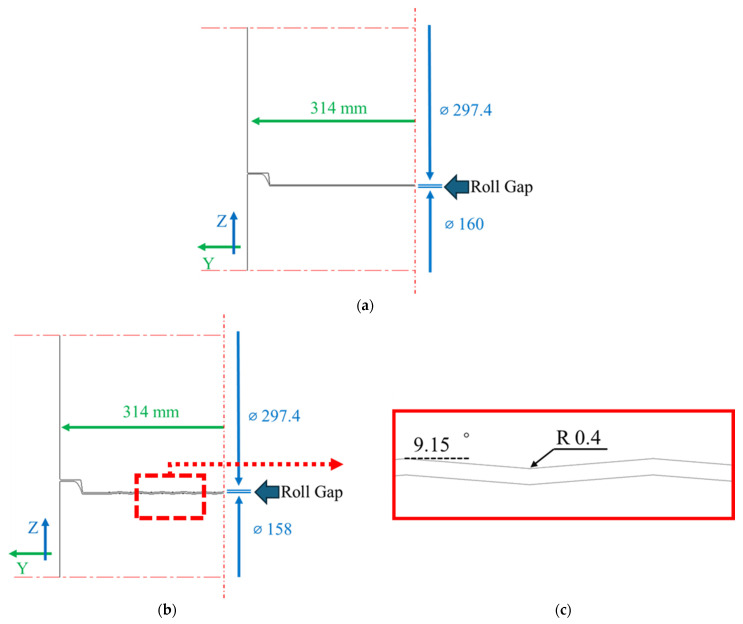
Configuration of roll stations at (**a**) the first-stage and third-stage rolls and (**b**) the second-stage roll. (**c**) Shape of the second-stage roll.

Figure 5 provides a detailed representation of each roll station. Figure 5a shows the configuration of the first roll and third roll, which are composed of flat rolls. Figure 5b depicts the configuration of the second roll, which features a shape distinct from that of the first and third rolls. This unique shape is shown in greater detail in Figure 5c.

Figure 5 shows the geometry of the second roll, defined by a slope angle of 9.15° and a radius of *R*0.4, which contrasts with the flat roll design of the first and third rolls. The design of the second roll focuses on improving flattening in the transverse direction (y in Figure 5a), rather than the longitudinal direction typically addressed in roller flattening processes. The slope and curvature are adjusted to leverage the alternating tension and compression effects inherent to the flattening process, redistributing residual stress across the width of the sheet. The metal sheet used in this experiment measured 232.2 × 1100 × 1.3 mm and was intentionally selected with a twist defect to evaluate the flattening performance. The roll gap was initially set to the sheet thickness of 1.3 mm and then progressively reduced to 1.1 mm, 0.9 mm, and 0.7 mm, with each configuration being tested three times. This systematic variation in roll gap allowed for the analysis of its effect on flattening efficiency.

### 2.3. Measurement Method

**Figure 6:**
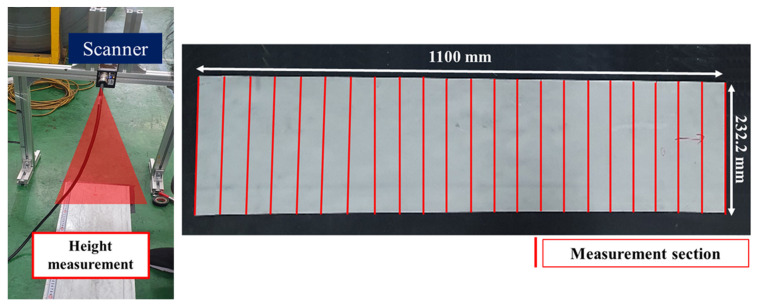
Scanning method and example of a measurement section.

The data was measured using a line scanner, as shown in Figure 6. A Keyence LJ-X8400 scanner (Osaka, Japan) was placed above the specimen and used a laser to scan and measure the height with high accuracy. This scanner has a resolution of 3.2 µm in the X direction and 0.2 µm in the Z direction, with a measurement accuracy of σ ≤ 0.08 µm, ensuring very small errors. The scanner measured the height from a fixed position, covering the entire specimen without needing to move. To keep the measurements accurate and consistent, all tests were done on a flat surface.

To check the reliability of the measured data, the standard deviations of crossbow and twist defect reductions at different roll gaps were calculated. Table 2 shows these results, helping to understand how much the defect reduction varies depending on the roll gap. The standard deviation of crossbow reduction was very low at a 1.1 mm roll gap (0.002 mm), meaning the measurements were very consistent. However, at 0.9 mm (0.326 mm) and 0.7 mm (0.245 mm) roll gaps, the variation was larger, showing that the measurement values changed more as the roll gap got smaller.

For twist reduction, the standard deviation also changed with different roll gaps. The lowest variation was at 1.1 mm (0.356 mm), while the highest was at 0.7 mm (0.730 mm). This pattern suggests that as the roll gap gets smaller, the material bends more, making the twist reduction values less stable. The higher strain and stress at smaller roll gaps can make small defects worse and cause more changes in the results. Even though there is some variation, the overall trend in defect reduction remains steady, confirming that the measured data is reliable.

As shown in Figure 6, twenty-two measurement points were selected along the width (1100 mm) and length (232.2 mm) of the specimen. These points were positioned at regular intervals to ensure that data from different areas of the surface could be analyzed effectively. A measurement interval of 50 mm was chosen to provide an optimal balance between capturing detailed deformation features and maintaining computational efficiency.

This interval was determined to be sufficient for capturing critical deformation patterns such as twist and crossbow defects while avoiding excessive computational burden. By comparing the height values at these locations, the flattening effect could be evaluated, and differences between processing conditions could be examined. This approach ensures that local variations in sheet deformation are properly analyzed without introducing unnecessary data complexity. 

However, it should be noted that the rolling force was not directly measured in this study, which may raise concerns regarding the reproducibility of the results. Since rolling force significantly influences the deformation behavior of the sheet, the absence of direct force measurements may present challenges in accurately validating the simulation results and comparing them with experimental outcomes.

The direct measurement of rolling force poses considerable challenges due to limitations in equipment and experimental constraints. The roll flattening process occurs over a very short duration under high compressive forces, making it difficult to obtain direct stress or force measurements with high accuracy. Furthermore, the second roll has a small curvature radius (R = 0.4 mm), resulting in a highly localized contact area. This leads to significant concentration of local stress, which poses challenges for strain measurement through surface patterns. Conventional optical strain measurement techniques, such as Digital Image Correlation (DIC), may not be suitable in such conditions, as excessive local deformation and non-uniform strain distribution can affect pattern visibility and reduce measurement accuracy.

**Figure 7:**
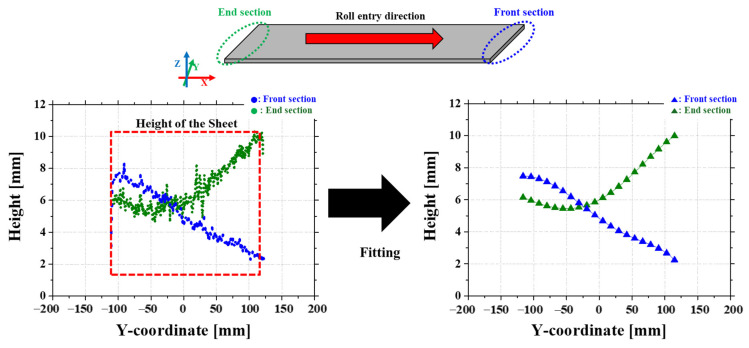
Smoothing of measured data through fitting.

To reduce measurement noise, a polynomial fitting method was applied to smooth the data. This approach ensured a consistent trend across the dataset, making it suitable for assessing the flattening results and identifying any remaining surface irregularities. As illustrated in Figure 7, the front and end sections are shown as examples to show the effect of the smoothing process. The scanning method provides high-resolution height data, allowing for the detection of small surface variations, such as distortions caused by residual stress.

The provided data corresponds to the flattening experiment before rolling at a roll gap of 1.1 mm. The X values range from −116.28 mm to 116.04 mm. A 5th-degree polynomial regression was applied to each dataset to approximate the measured values. For the front section dataset, the polynomial regression equation is as follows:

(2) $$f_{1}\left(x\right)=4.23\times 10^{-12}x^{5}-3.92\times 10^{-9}x^{4}+5.88\times 10^{-8}x^{3}-5\times 10^{-5}x^{2}-0.0199x+8.41,$$

The coefficient of determination ($R^{2}$) is 0.940, and the standard deviation (RMSE) is 0.363.

For the end section dataset, the polynomial regression equation is as follows:

(3) $$f_{1}\left(x\right)=4.12\times 10^{-12}x^{5}-3.85\times 10^{-9}x^{4}+5.92\times 10^{-8}x^{3}-4.85\times 10^{-5}x^{2}-0.0192x+8.35,$$

The coefficient of determination ($R^{2}$) is 0.982, and the standard deviation (RMSE) is 0.221. The fitted data for the two values is shown in Figure 3.

Since the same processing method was applied uniformly to all measurement points, the results remain comparable across different specimens and processing conditions.

## 3. Methods

### 3.1. Analysis Model

**Figure 8:**
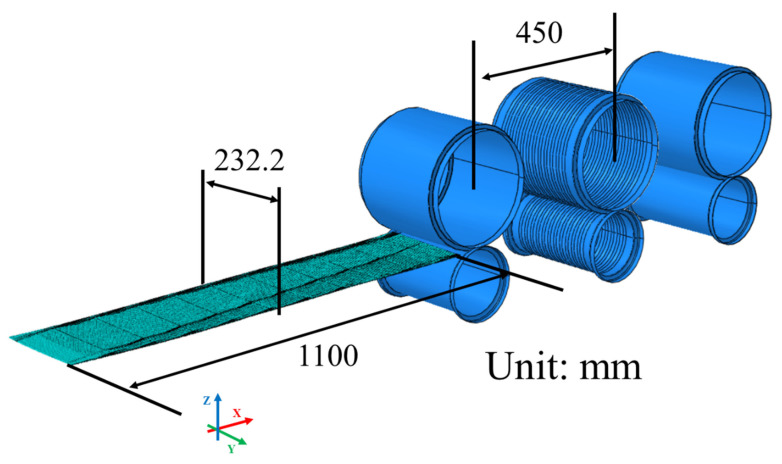
Arrangement of rolls and sheets used in the simulation.

The simulation roll setup shown in Figure 8 is made up of three rolls. The first roll and third roll have a traditional flat shape, but the second roll is designed with a repeated pattern (see Figure 5). Conventional leveler rolls mainly work by applying repeated tension and compression along the sheet’s length (X direction) to lessen residual stress and flatten the sheet [5,16]. In this study, however, we use the shape of the second roll to change the same tension-compression cycle to the sheet’s width (Y direction), which effectively reduces uneven local residual stress [6,9]. 

The local deformation in the width direction from the second roll is adjusted by the nearby lower third roll. The area that is stretched in the second roll is compressed by the flat roll of the third roll, while the area that is compressed in the second roll is stretched by the third roll, restoring the overall flatness of the sheet. This method follows the same tension-compression cycle as the traditional roll flattening method but, by switching the direction of the force, it can more precisely control the residual stress across the sheet’s width [6]. This approach helps solve problems with uneven residual stress that may occur during the sheet-making process and greatly improves the uniformity of the final shape, as confirmed by both numerical analysis and experiments [17]. 

The analysis model was developed using an analytic rigid body based on the roll drawings, and the distance between rolls was set to 450 mm. The sheet was modeled with shell elements, and the contact condition between the rolls and the sheet was set with a friction coefficient of 0.12. In addition, an initial speed of 200 mm/s was applied to the sheet, with the angular speeds (w = v/r) of the rolls set as follows: the upper roll at −1.345 rad/s, lower rolls 1 and 3 at 2.5 rad/s, and the lower second roll at 2.53 rad/s to account for a small difference in diameter. Under these conditions, the analysis shows that the shape of the second roll plays a key role in flattening the sheet.

To account for roll elastic deformation, we modeled the bottom roll as a rigid fixed body while the upper roll was supported by a spring to approximate its elastic behavior under load. This approach is supported by previous studies, which have demonstrated that a spring-supported rigid roll model effectively captures roll deformation effects while maintaining computational efficiency [18]. Adjusting the spring stiffness enables accurate shape predictions without requiring a fully elastic roll model, which would significantly increase computational cost. Furthermore, studies by [19,20] indicate that while rigid roll models may introduce some errors in force and stress predictions, they are generally sufficient for shape accuracy in ultra-high-strength steel (UHSS) applications. Our numerical analysis results confirm that the calibrated spring-supported rigid roll model provides shape prediction accuracy comparable to experimental results while reducing computational cost.

### 3.2. Analysis Conditions

#### 3.2.1. Comparison Between Solid and Shell Elements

A comparative analysis was conducted between shell and solid elements under identical mesh and boundary conditions to assess their suitability for simulating the flattening process. Both models utilized the same mesh structure, with the height in the z-direction treated as an integration point to ensure consistency in the evaluation. The primary objective of this comparison was to determine how accurately each element type could represent roll geometry and its interaction with the sheet. The findings showed a clear limitation of the shell element model.

**Figure 9:**
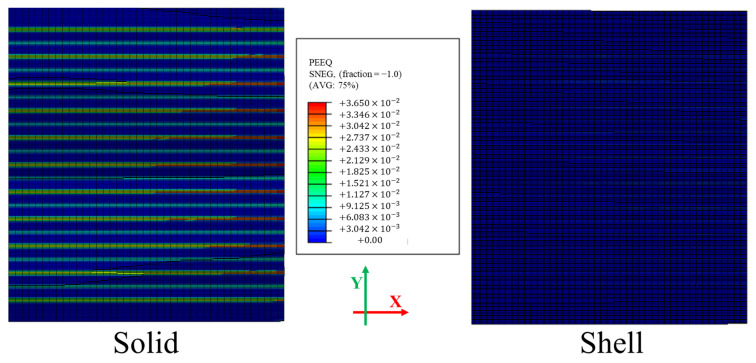
Comparison of equivalent plastic strain (PEEQ) distribution between solid and shell models.

As shown in Figure 9, which depicts the equivalent plastic strain (PEEQ) distribution after the flattening process, the shell model failed to capture the influence of roll geometry on the sheet [21]. Unlike the solid model, the shell model did not properly account for the force exerted by the rolls, leading to an inaccurate representation of local stress and strain distributions. This discrepancy arises from the structural limitations of shell elements, which inherently assume a plane stress condition and neglect through-thickness stress variation. As a result, shell elements are unable to represent the actual load transfer through the thickness direction, resulting in an inaccurate PEEQ distribution.

In contrast, the solid element model effectively incorporated the detailed roll geometry and accurately simulated the three-dimensional load transfer from the rolls to the sheet. This enabled it to capture the actual deformation and stress distribution, including through-thickness normal and shear stresses, leading to a more realistic representation of the flattening process. The rolling-induced deformation is characterized by large in-plane stress variations and heavily constrained out-of-plane strain, making the deformation state of the sheet better represented by a plane strain condition rather than a plane stress condition. The significant through-thickness compressive stresses generated by the rolls further support this assumption. Therefore, the solid element model, which does not impose any restrictive stress assumptions, offers a more suitable and accurate framework for analyzing the flattening behavior. Given its superior performance in replicating physical behavior and capturing stress redistribution, the solid model was ultimately adopted for the analysis.

#### 3.2.2. Number of Elements

The total number of elements used in the simulation was approximately 153,120 (X: 220; Y: 232; Z: 3). The element size in the X direction (rolling direction) was set to 5 mm, dividing the total length of 1100 mm into 220 elements. In the Y direction (transverse direction), an element size of 1 mm was applied, resulting in 232 elements across a width of 232.2 mm. The Z direction (thickness direction) was discretized with three elements to capture the sheet’s 1.3 mm thickness accurately. Among these, the Y direction element size had the greatest influence on simulation accuracy. While the X direction represents the relative movement between the rolls and the sheet, variations in element size along this axis have a relatively minor impact on contact accuracy. Similarly, in the Z direction, an adequate number of elements is necessary to model contact pressure and strain distribution, but explicit simulations remain stable with as few as three elements. In contrast, the Y direction plays a crucial role in defining the roll curvature and is the key factor in determining the accuracy of the contact simulation. Increasing the number of elements in the X or Z direction significantly raises computational costs due to the reduction in time step size in explicit analysis. Since the time step is governed by the smallest element size, excessive refinement in these directions would dramatically increase computation time without a proportional gain in accuracy. Therefore, optimizing the Y direction element size while maintaining practical element counts in the X and Z directions provided the best balance between accuracy and computational efficiency.

**Figure 10:**
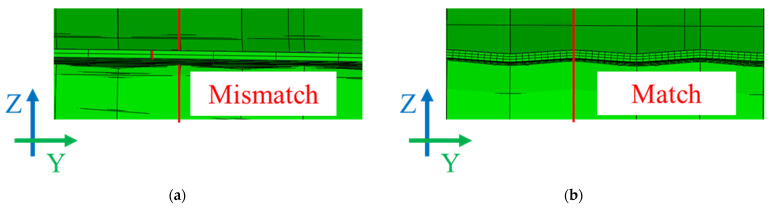
Effect of Y direction element size on roll geometry representation: (**a**) element size 10 mm (mismatch); (**b**) element size 1 mm (match).

Figure 10 shows the effect of the Y direction element size on the accuracy of the roll geometry representation in the simulation. When the Y direction element size was 10 mm, the red line representing the second roll’s inclined pattern did not align with the sheet mesh structure, indicating that the roll pattern was not properly reflected in the deformation. This discrepancy led to errors in transverse deformation representation, making it impossible to evaluate the effect of the second roll accurately.

Conversely, when the Y direction element size was reduced to 1 mm, the red line perfectly matched the roll pattern, confirming that the roll geometry was accurately captured in the simulation. Given that transverse deformation is a crucial factor, as emphasized in the introduction, reducing the Y direction element size is essential for accurately analyzing the effect of the second roll. Furthermore, Figure 5c indicates that the roll pattern has an R value of 0.4 mm, suggesting that a finer mesh in the Y direction is required to properly reflect the actual roll geometry. 

The analysis systematically examined the boundary conditions, element types, mesh configuration, and contact modeling approach in the FEM simulations. The impact of Y direction element size on contact accuracy was analyzed in detail using Figure 10, leading to the selection of the optimal element size. As shown in Figure 10, when the Y direction element size was 10 mm, the roll pattern and sheet mesh did not align, reducing the reliability of the contact simulation. However, with a 1 mm element size, the roll pattern was accurately reflected in the sheet deformation, improving the precision of the simulation results.

Since transverse deformation is particularly important and is primarily influenced by the second roll, minimizing the Y direction element size is essential to ensure an accurate representation of the roll-sheet interaction. Additionally, as shown in Figure 5c, the roll pattern has an R value of 0.4 mm, further emphasizing the need for a finer Y direction mesh for greater accuracy. Based on these findings, the optimal Y direction element size was determined to be 1 mm, ensuring strong agreement between the simulation and experimental results.

#### 3.2.3. Spring Stiffness

To improve the accuracy of the analysis and better model the interactions between the sheet and the rolls, this study introduced spring stiffness as a key variable to account for elastic deformation effects. In practical roll flattening processes, the contact between the sheet and the rolls is influenced by local elastic deflection of the rolls, which affects the load transfer and overall flattening performance. Previous studies [22] have shown that incorporating elastic compliance into roll models can significantly reduce discrepancies between experimental and numerical results. 

To determine the optimal spring stiffness, an experimental calibration approach was employed. The initial stiffness was set to 10 N/mm and progressively increased in steps of ten to examine the deformation behavior under two critical conditions: the widest roll gap of 1.3 mm and the narrowest of 0.7 mm. Specifically, in the second roll, the optimal spring stiffness was determined by noting the state just before excessive marking began at the 1.3 mm gap and by finding the critical point when marks formed at the 0.7 mm gap. The experimental verification showed that a spring stiffness of $10^{5}$ N/mm behaved most like the experimental data, and this value was chosen as optimal. This result not only supports the model’s validity but also shows its potential for use in actual processes. In addition, while the lower roll was kept fixed, the three upper rolls were allowed to move freely in the z-direction, with all degrees of freedom except rotation around the y-axis being restricted. With this setup, spring stiffness was applied to the upper rolls to accurately model the elastic interaction between the rolls and the sheet. This allowed for a more precise analysis of roll deflection and contact forces. 

In summary, introducing spring stiffness significantly enhanced the model’s accuracy and reliability, particularly in capturing roll deflection effects that impact local deformation and stress redistribution. By incorporating a calibrated spring stiffness value, the simulation better approximates experimental conditions, ultimately improving predictive accuracy in roll flattening simulations.

#### 3.2.4. Contact and Dynamic Influences

In this analysis, the effects of contact pressure and friction were considered by setting the friction coefficient to 0.12 because the experimental setup involved a lightly oiled metal sheet surface, which typically results in a friction coefficient ranging from 0.10 to 0.15 [23]. The median value of 0.12 was chosen to represent this behavior accurately. Additionally, a combination of general contact with hard contact conditions was used to effectively model the interaction between the sheet and the rolls, ensuring that the contact pressure is accurately captured in the simulation. 

The frictional stress (*τ* = *μ*) is highly sensitive to variations in the friction coefficient (*μ*), especially as the reduction per pass increases. Experimental studies have shown that the friction coefficient is not constant along the roll gap and varies with rolling speed, reduction, and lubrication conditions [24,25]. In rolling processes, pressure fluctuations are mitigated through elastic roll deformation or lubrication adjustments, whereas simulations typically assume rigid rolls and a constant friction coefficient, leading to discrepancies [25]. Explicit simulations also use techniques such as mass scaling (set to 1000 in this study) and artificially increased process speeds to save computing time. However, these methods can amplify inertial effects, causing unrealistic stress wave propagation and dynamic oscillations when the sheet meets the rolls. These effects have been widely studied in the context of explicit finite element simulations for sheet forming, showing that the lack of damping effects from bearings, stand structures, and frictional interfaces can lead to overestimated deformation predictions [26]. Since explicit methods rely on dynamic time integration without inherent energy dissipation mechanisms, exaggerated stress fluctuations and deformation patterns can occur, causing further deviations from experimental results [26]. 

As a result, while this study applies the analysis conditions, differences between simulation and experimental rolling conditions may lead to discrepancies in the results.

## 4. Results and Discussion

### 4.1. Comparison of Sheet Geometry and RMSE for Different Gaps

**Figure 11:**
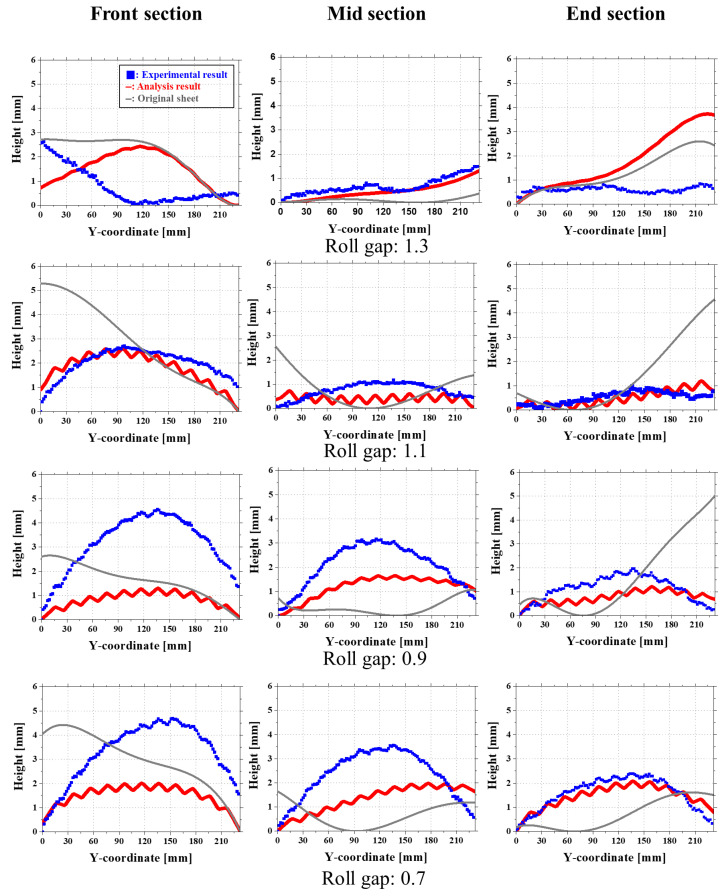
Comparison of experimental (blue), simulated (red), original sheet (gray) cross-sectional profiles under different gap conditions (1.3 mm, 1.1 mm, 0.9 mm, 0.7 mm).

**Figure 12:**
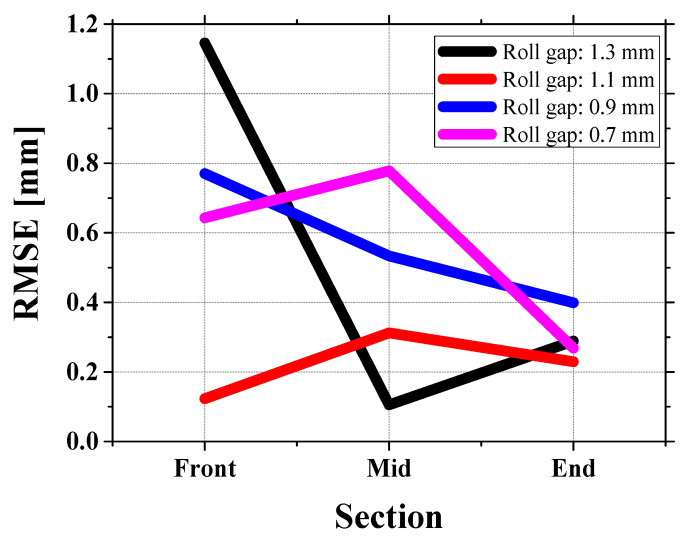
Comparison of RMSE by gap setting and section position.

This study analyzed cold roll forming under four gap conditions: 1.3 mm, 1.1 mm, 0.9 mm, and 0.7 mm through experiments and finite element simulations using Abaqus Explicit (version 2024, Dassault Systems, Johnston, RI, USA), where Figure 11 compares the cross-sectional profiles of the sheet (blue: experiment, red: simulation, gray (original sheet) in three sections (front, mid, end), and Figure 12 summarizes the RMSE for each section. 

**Figure 13:**
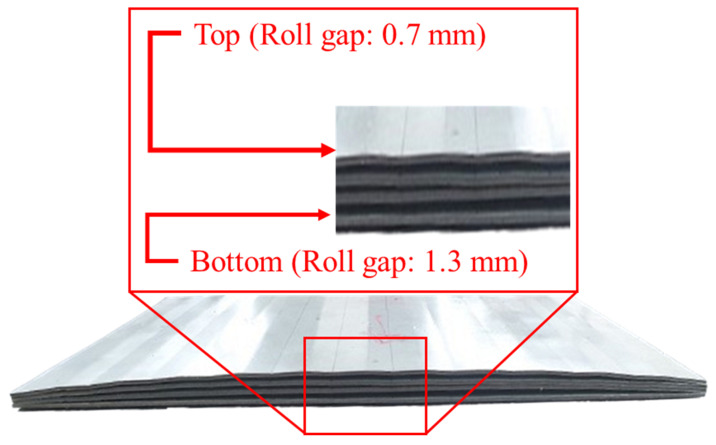
Sheets arranged in descending roll gap order (0.7 mm → 1.3 mm) to illustrate post-flattening deformation trends.

To further examine the experimental deformation trends, Figure 13 provides a visual representation of the post-flattening height changes for each roll gap condition. The metal specimens are arranged in descending roll gap order (0.7 mm → 0.9 mm → 1.1 mm → 1.3 mm) to highlight the differences in crossbow deformation. This side view of the specimens allows for a clearer comparison of the effect of roll gap reduction on sheet flattening.

**Figure 14:**
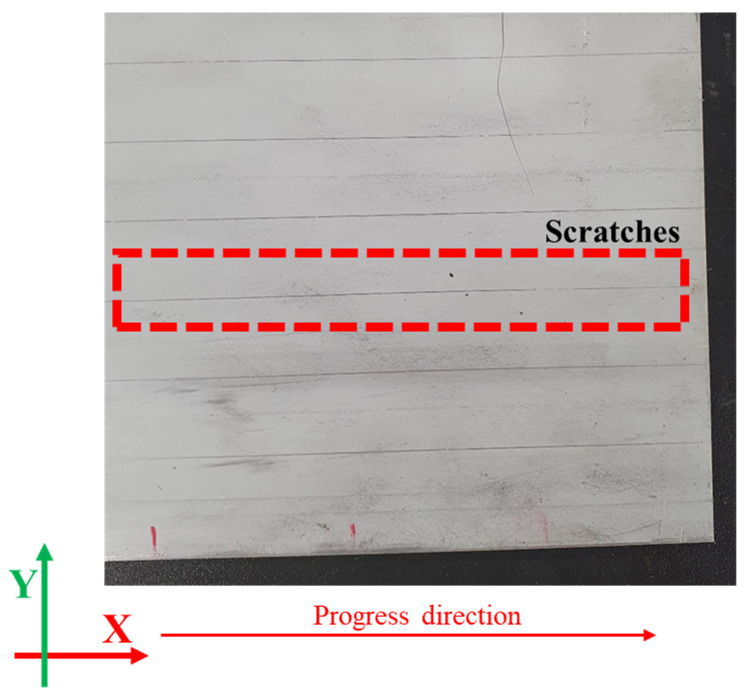
Surface scratches on the sheet caused by the second roll at a 1.1 mm roll gap.

At a 1.3 mm roll gap, the average RMSE is approximately 0.51 mm. On the other hand, at a 1.1 mm gap, the RMSE falls between 0.12 and 0.31 mm, indicating better agreement between experiments and simulations. However, examining the surface marks on the second roll reveals a key issue. In the experiments, no noticeable marks were observed at the 1.3 mm gap, while small but distinct marks appeared on the sheet surface at the 1.1 mm gap (see Figure 14).

In contrast, the simulations show that at the 1.3 mm gap, a thin roll mark with a front depth of approximately 0.1 mm is present, whereas a more pronounced roll mark appears at the 1.1 mm gap. These discrepancies may be attributed to stress concentration, material recovery properties, friction effects, and simulation settings. The second roll, featuring an inclined repeating pattern at an angle of 9.15°, induces a small height variation of approximately 1.59 mm (see Figure 5c). Additionally, the tip of the second roll is very sharp (0.4 R), leading to significant local stress concentration on the sheet surface due to periodic changes in contact pressure. 

The study also finds that the simulation overestimates the second roll’s effect at a roll gap of 1.3 mm, leading to a reversed crossbow. While this may stem from inaccuracies in friction or force distribution, no friction sensitivity analysis was conducted. Performing simulations with varying friction coefficients (e.g., 0.10 to 0.15) would help assess the variability and improve the robustness of the model.

If the mesh size were very small (matching the width of the experimental scratch marks), the phenomenon could be simulated more accurately. However, since the mesh size is set to 1 mm, the overall roll mark effect appears exaggerated. Further reducing the mesh size would significantly impact the explicit time step, making it difficult to implement in the simulation. Moreover, since the simulation models the roll as a rigid body, any potential deformation of the roll that might help distribute the pressure is not captured, leading to more pronounced roll marks in the simulation. 

**Figure 15:**
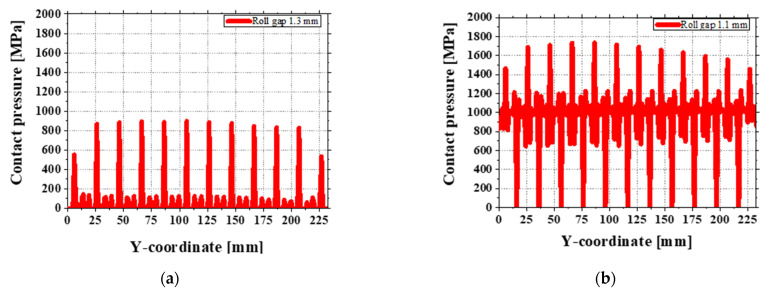
Contact pressure distribution along the Y-coordinate at (**a**) 1.3 mm and (**b**) 1.1 mm roll gaps.

At a 1.3 mm gap, the measured contact pressure at the front of the sheet is about 900 MPa (see Figure 15a), which is lower than the material’s yield strength. However, the small stress changes caused by the roll shape may trigger slight plastic deformation in the simulation, making the roll marks appear worse than they are. In contrast, at a 1.1 mm gap, the average contact pressure is higher, around 1400 to 1800 MPa (see Figure 15b). This higher pressure makes plastic deformation more likely, and some roll marks were observed in the experiments.

**Figure 16:**
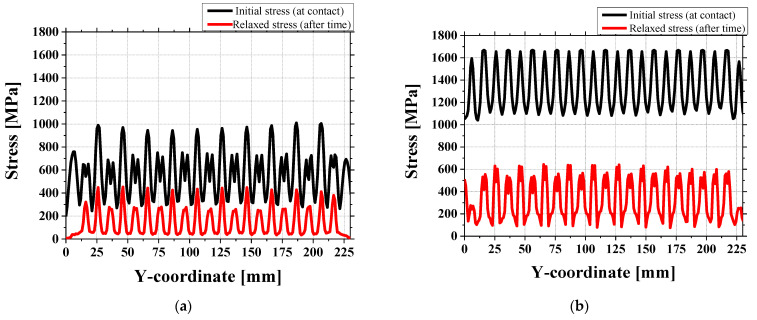
Comparison of von Mises stress distributions at initial contact and after stress relaxation (**a**) 1.3 mm and (**b**) 1.1 mm roll gaps.

Furthermore, to evaluate the time-dependent behavior of stress in the contact region, a stress relaxation analysis was conducted using finite element simulation. Figure 16 compares the von Mises stress distribution at the moment of contact (black line) and after a certain period (red line).

At the time of contact, the von Mises stress reached up to 1200–1600 MPa at the 1.1 mm roll gap, clearly exceeding the material’s yield strength and indicating the possibility of localized plastic deformation. However, as time progressed, a significant reduction in the stress magnitude was observed in the contact area. This stress relaxation suggests that the initially concentrated stress was redistributed due to plastic flow and material recovery effects.

The simulation results imply that although high stress levels may initially trigger plasticity, the actual residual stress may be much lower after stabilization, which helps explain the milder surface marks observed in experiments compared to simulations.

At a 0.9 mm gap, the RMSE increases to between 0.39 and 0.77 mm, and at a 0.7 mm gap, it reaches between 0.2683 and 0.77 mm. These values show a larger difference compared to the 1.1 mm gap. This indicates that as the roll gap gets smaller, the difference between the experiments and the simulation grows, especially in the front section where height differences (i.e., crossbow height differences) occur. The issue with the crossbow will be discussed in the next section.

### 4.2. Crossbow Defect

**Figure 17:**
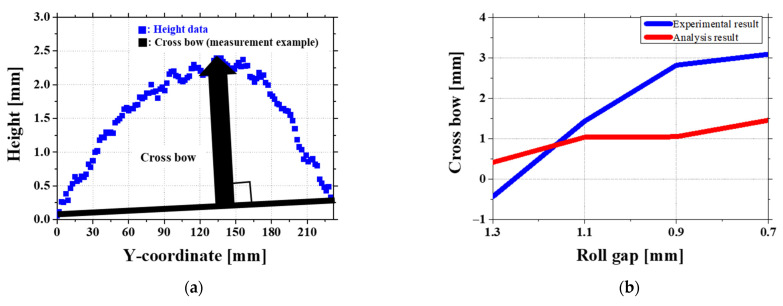
Experimental and simulation comparison of the crossbow under different roll gaps: (**a**) measurement method of the crossbow; (**b**) crossbow variations at different roll gaps.

Figure 17a shows the method used to measure crossbow using the central arch height relative to the straight edges of the sheet as a key indicator. Crossbow is an important shape defect in roll forming because too much arching can create problems in later processing steps. Figure 17b presents the measured crossbow values under four different roll gap conditions.

At a 1.1 mm gap, the experimental and simulation results are quite similar. The experiment recorded a crossbow height of 1.4306 mm, while the simulation estimated it at 1.0359 mm. This small difference suggests that the contact pressure and other process settings are well balanced at a 1.1 mm gap. 

**Figure 18:**
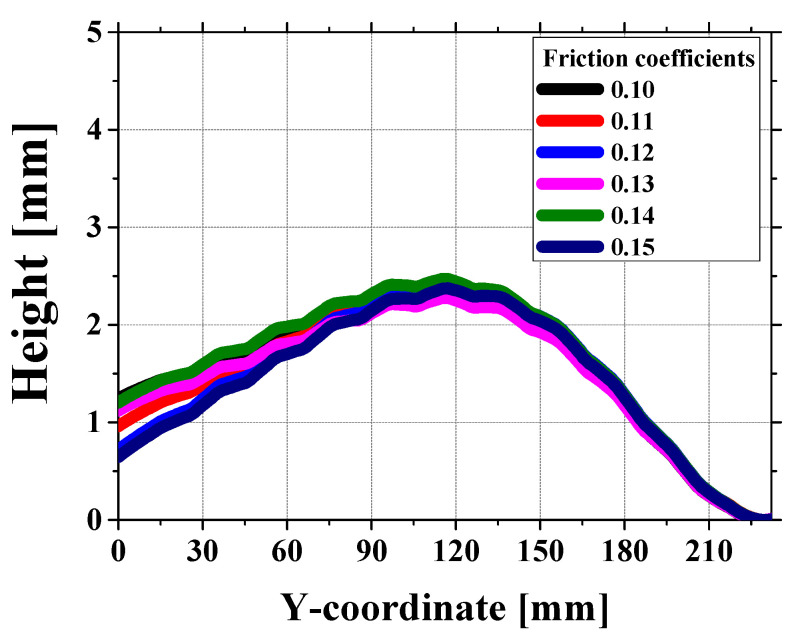
Height variation at the front section for roll gap 1.3 mm with different friction coefficients.

At a 1.3 mm gap, the crossbow shape in the simulation does not match the experiment. In particular, the front section shows a reversed crossbow pattern in the simulation, while this is not seen in the experiment. One possible reason is that the second roll does not have as much effect on equipment, whereas in the simulation, its influence is too strong, causing stress to build up in specific areas and reversing the crossbow shape. Also, the first contact area where the sheet touches the rolls is very sensitive to small changes, which may contribute to the difference [27]. As shown in Figure 18, the variation in friction coefficient from 0.10 to 0.15 does not lead to significant changes in the height profile along the Y-coordinate. This indicates that friction alone is not the dominant factor influencing the reversed crossbow formation. Instead, further investigation into stress concentration and boundary conditions is required to better understand the underlying causes.

At 0.9 mm and 0.7 mm gaps, the difference between the experiment and simulation becomes even larger. For example, at a 0.9 mm gap, the experiment recorded a crossbow height of 3.2742 mm, but the simulation predicted only 1.0492 mm. In the experiment, the crossbow gets much bigger as the roll gap gets smaller. However, in the simulation, the increase is much slower. This happens because the way the rolls are fixed and controlled is different in actual machines compared to computer models. In experimental, the ends of the rolls are firmly held in place by a shaft, which means the edges do not move much, but the center is free to bend more. Because of this, the force is not spread evenly across the width of the roll, making the crossbow much stronger in the experiment [28]. In the simulation, the rolls are treated as rigid bodies with a fixed spring stiffness. This means the force from the rolls is applied evenly across the sheet, so there is less pressure in certain areas. Because of this, the simulation predicts a much smaller increase in crossbow compared to the experiment.

Overall, the experiment always shows a bigger crossbow than the simulation. This is because the way the rolls are fixed and controlled affects how force is applied, and this effect is not fully included in the simulation. As the roll gap gets smaller, the uneven force distribution caused by the roll fixation becomes stronger, leading to a bigger gap between the experimental and simulated crossbow results.

### 4.3. Twist Defect

**Figure 19:**
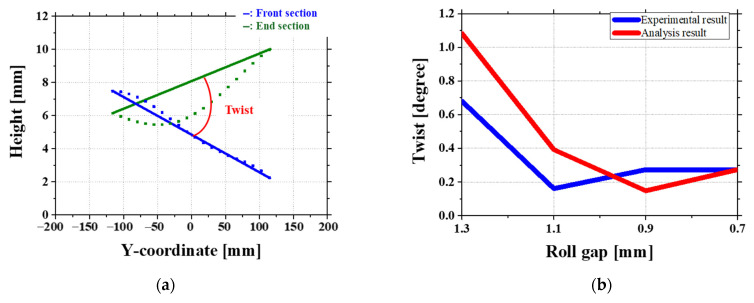
Experimental and simulation comparison of twist defects under different roll gap conditions: (**a**) twist measurement method; (**b**) twist variation across roll gaps.

Figure 19a also shows the method used to measure twist. Twist is defined as the difference in angle between the front and end of the sheet and is one of the key defects in roll forming. It is strongly affected by uneven stress along the sheet length. Figure 19b compares the twist results from experiments and simulations under four different roll gap conditions.

At roll gaps, both the experimental and simulation results for twist exhibit an initial reduction followed by a subsequent increase, as shown in Figure 19b. In the experimental results, twist decreases up to a roll gap of 1.1 mm and then starts to increase from 0.9 mm. In the simulation results, twist continues to decrease until 0.9 mm and then increases at 0.7 mm. Twist is calculated as the angular difference between the front and end tilt of the sheet. This variation in twist originates from the stress difference between the front and end sections of the sheet. 

**Figure 20:**
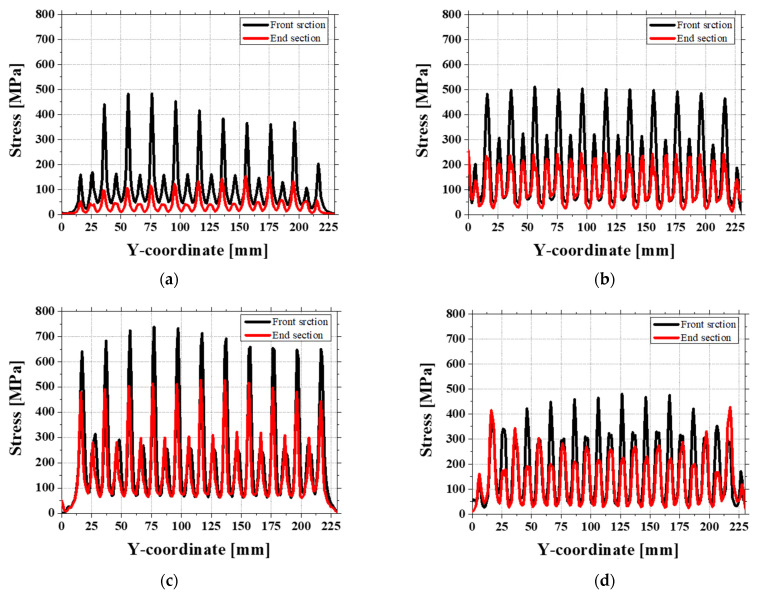
Stress difference range for front and end sections at roll gaps of (**a**) 1.3 mm, (**b**) 1.1 mm, (**c**) 0.9 mm, and (**d**) 0.7 mm.

**Figure 21:**
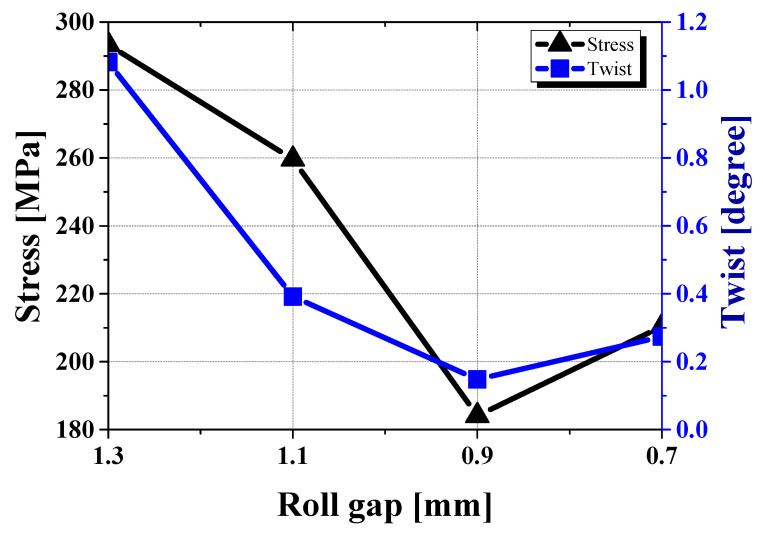
Stress differences and twist changes with roll gap.

Therefore, after each roll gap analysis, the stress difference between the front and end sections is compared [29]. Special attention is given to the region influenced by the second roll, as indicated in Figure 20. The stress ranges at different roll gaps are as follows: 1.3 mm gap: 209.995–376.647 MPa; 1.1 mm gap: 248.275–270.907 MPa; 0.9 mm gap: 142.637–225.554 MPa; 0.7 mm gap: 166.053–254.638 MPa. By using the midpoints of these stress ranges to generate a graph, a trend like the twist variation in the simulation is observed, as shown in Figure 21. This suggests that the twist is primarily driven by the stress imbalance between the front and end sections of the sheet. The particularly low twist observed at the 0.9 mm gap in the simulation can be explained by the preceding crossbow formation. The contact between the sheet and the roll in the front region plays a significant role, where a lower roll gap increases the contact effect, reducing the stress imbalance between the front and end sections. As a result, both twist and crossbow defects improve. This also explains why the simulation results predict lower twist values than the experimental results at this specific roll gap. 

Overall, the relationship between twist and stress imbalance shows the importance of front-end stress distribution in sheet forming. The results show that as the roll gap decreases, front-end contact effects become more pronounced, leading to a more balanced state and a reduction in twist, especially at specific gaps such as 0.9 mm.

## 5. Conclusions

This study investigates a three-stage roll flattening process for 1.5 GPa grade AHSS, comparing experimental and FEM results under various roll gap conditions. It focuses on a unique transverse deformation mechanism induced by the second roll, which differs from conventional longitudinal flattening methods.

Key findings include the following:

- A 1.1 mm roll gap was found to be the most effective condition, reducing twist defects by more than 80% and minimizing crossbow deformation (RMSE ≈ 0.22 mm).
- At a 1.3 mm gap, simulations overestimated the effect of the second roll, leading to a reversed crossbow pattern in the front section, which was not observed in experiments.
- When the roll gap was reduced to 0.9 mm and 0.7 mm, the experiment showed a significant increase in crossbow deformation, while the simulation underestimated this effect due to limitations in considering friction changes and local stress accumulation. In experimental, the end of the roll is fixed by a shaft, limiting the edge movement, resulting in a more bent center, resulting in a disproportionate force distribution and stronger crossbow deformation. On the other hand, the simulation lowers the crossbow prediction by evenly applying force throughout the sheet. Due to this difference in roll fixation, the experiment consistently shows a larger crossbow than the simulation, and this effect becomes more pronounced as the roll gap decreases.

These findings confirm that deformation behavior is strongly influenced by frictional changes and local stress accumulation, factors that are only partially considered in numerical models. Furthermore, optimizing the shape of the second roll and adjusting the roll gap can significantly reduce the need for additional leveler rolls while improving overall flattening performance. These insights contribute to enhancing the roll flattening process for high-strength steel sheets, thereby improving both production efficiency and product quality.

## Figures and Tables

**Table 1:** Mechanical properties and Voce hardening parameters (unit: MPa).

Parameter	Value
Young’s modulus	217,000
Poisson’s ratio	0.33
** $\sigma_{y}$ **	1251.73
** $n_{1}$ **	392.74
** $n_{2}$ **	419.64
** $n_{3}$ **	500

**Table 2:** Standard deviations of twist and crossbow defects at different roll gaps.

Roll Gap (Unit: mm)	Twist (Unit: Degree)	Crossbow (Unit: mm)
1.3	0.532	0.138
1.1	0.356	0.002
0.9	0.481	0.326
0.7	0.730	0.245

## Data Availability

The original contributions presented in this study are included in the article. Further inquiries can be directed to the corresponding author.
